# Characterization of insecticide resistance mechanisms in the *Anopheles gambiae* population of Sierra Leone

**DOI:** 10.1186/s12936-025-05267-z

**Published:** 2025-03-13

**Authors:** Kevin Ochieng’ Opondo, Evelyne Alyko, Samuel Smith, Rebecca Levine, Jenny Donnelly, Tony Hughes, David Schnabel, Ramlat Jose, Alpha Jalloh, Umaru Lolleh, Samuel Conteh, Laurent Iyikirenga, Djenam Jacob, Miriam Mokuena, Frederick Yamba, Yemane Yihdego

**Affiliations:** 1 US President’s Malaria Initiative Evolve Project, Abt Associates, Freetown, Sierra Leone; 2 National Malaria Control Programme, Ministry of Health and Sanitation, Freetown, Sierra Leone; 3 US Centers for Disease Control and Prevention, Atlanta, USA; 4 US President’s Malaria Initiative, USAID, Washington, DC USA; 5 US Navy and Marine Corps Public Health Center Detachment, Centers for Disease Control and Prevention, Atlanta, USA; 6 US President’s Malaria Initiative, Centers for Disease Control and Prevention, Freetown, Sierra Leone; 7 US President’s Malaria Initiative, US Agency for International Development, Freetown, Sierra Leone; 8 US President’s Malaria Initiative Evolve Project, Abt Associates, Rockville, MD USA; 9 School of Community Health Sciences, Njala University, Bo, Sierra Leone

**Keywords:** Insecticide resistance, *Anopheles gambiae*, *Kdr*, *Ace-1*, Sierra Leone

## Abstract

**Background:**

Information on the status of insecticide resistance in malaria vectors is critical for implementing effective malaria vector control. The Sierra Leone National Malaria Control Programme, in collaboration with the PMI VectorLink project, assessed the resistance status to insecticides commonly used in public health, and associated resistance mechanisms in *Anopheles gambiae*, the main vector of malaria in Sierra Leone.

**Methods:**

The susceptibility of *An. gambiae* against pyrethroids with and without piperonyl butoxide (PBO), chlorfenapyr, clothianidin, bendiocarb and pirimiphos-methyl was evaluated in four districts of Sierra Leone in 2018 and 2019 using WHO and CDC bottle bioassay protocols. A subset of samples that were exposed to the insecticides were screened for molecular markers of insecticide resistance, *knock-down resistance (kdr)* L1014F, 1014S and N1575Y, and (*ace-1*-G119S).

**Results:**

*Anopheles gambiae* from all sites were resistant to the diagnostic doses of three pyrethroids: deltamethrin, permethrin and alpha-cypermethrin. Intensity of resistance to all three pyrethroids was high, with less than 95% mortality at 10X concentration. However, pre-exposure of *An. gambiae* to PBO increased overall mortality by 41.6%, 50.0% and 44.0% for deltamethrin, permethrin and alpha-cypermethrin, respectively. The vector was susceptible to chlorfenapyr, clothianidin and pirimiphos-methyl, while bendiocarb showed possible resistance. The frequency of *kdr* alleles was 98.2% for L1014F, 2.1% for 1014S and 8.9% for N1575Y, while the frequency of the *Ace-1* G119S allele was 13.6%. Significant deviation from the Hardy–Weinberg equilibrium and deficiency of heterozygotes was detected only at the G119S locus of *An. gambiae* (p < 0.0001)*.* Of the 191 *An. gambiae *sensu lato that were molecularly identified to the species level, 81.7% were *An. gambiae *sensu stricto (95% CI 75.3–86.7), followed by *Anopheles coluzzii* (17.8%, 95% CI (12.8–24.1) with one hybrid of *An. gambiae/An. coluzzii* 0.5%, 95% CI (0.03–3.3).

**Conclusion:**

Malaria vectors were highly resistant to pyrethroids but exposure to PBO partially restored susceptibility in *An. gambiae* s.l. in Sierra Leone. Malaria vectors were susceptible to chlorfenapyr, clothianidin and pirimiphos-methyl with possible resistance to bendiocarb. These data informed the selection and distribution of ITN PBO in Sierra Leone’s mass campaigns in 2020 and selection of clothianidin for indoor residual spraying in 2021.

**Supplementary Information:**

The online version contains supplementary material available at 10.1186/s12936-025-05267-z.

## Background

Globally, an estimated 247 million cases of malaria and 619,000 deaths occurred in 2021 with 95% of the cases reported from Africa [1]. The majority (96%) of malaria deaths occurred in Africa with children under 5 years of age accounting for 80% of the malaria deaths [1, 2]. Between 2000 and 2015, global malaria case incidence declined by 27%, and by less than 2% between 2015 and 2019, and stagnated since 2021 indicating a slowing of the rate of decline since 2015 [1, 3]. Indoor residual spraying (IRS) and insecticide-treated nets (ITNs) are considered core interventions in the global control of malaria, which have contributed to the decline in malaria cases globally over the last two decades [1, 3]. However, the intensive use of insecticides in agriculture and to a lesser degree in public health has led to widespread resistance among targeted mosquito vectors [4–6] and the major gains in the control of malaria have been threatened due to insecticide resistance [4].

Malaria is endemic in Sierra Leone, with stable and perennial transmission throughout the country. It is the leading cause of morbidity and mortality and accounts for 40.3% of all-age outpatient morbidity, 37.6% of hospitalizations, and has a case fatality rate of 17.6% in children under five years of age [7]. The Sierra Leone National Malaria Control Programme (NMCP) conducted mass distribution of ITNs in 2010, 2014, 2017 and 2020 to achieve universal coverage. The mass distribution in May 2020 was conducted solely with ITNs impregnated with a pyrethroid and the synergist piperonyl-butoxide (PBO ITNs), making Sierra Leone the world’s first country to deploy PBO ITNs nationwide. The next mass distribution campaign using PBO ITNs and Interceptor G2 (IG2, active ingredients of chlorfenapyr and alpha-cypermethrin) ITNs is planned to begin in January 2024. The mass campaigns are complemented by routine IG2 or PBO ITN distribution through antenatal care (ANC) and Expanded Programme on Immunization (EPI) clinics including during Maternal and Child Health Week. A pilot IRS campaign with lambda-cyhalothrin was implemented in four districts in 2011 and 2012, covering 5.8% of the population in Sierra Leone. In 2021, the NMCP in collaboration with the U.S. President’s Malaria Initiative (PMI) began implementing IRS with SumiShield™ (active ingredient, clothianidin) in Bo and Bombali districts that had the highest entomological inoculation rates (EIR) of over 410 infectious bites/person/year with malaria prevalence of between 38 and 40% in children under 5 years [7–9].

Successful implementation of a vector control programme relies on knowledge of vector species, behaviour, infectivity, and susceptibility to insecticides [10]. Members of the *Anopheles gambiae* complex and *Anopheles funestus* group are the major vectors of malaria in sub-Saharan Africa (SSA), including Sierra Leone [11, 12]. Prior to this study, *An. gambiae *sensu stricto (*s.s*.) and *Anopheles coluzzii* had been reported in Sierra Leone but their insecticide resistance status was not well established due to the disruption of entomological studies during the civil war (from 1991 to 2002) and beyond [13]. Characterization of insecticide resistance in mosquitoes was later reported, but only in urban Freetown [14]. The knock-down resistance *(kdr)* mutation conferring resistance to pyrethroids and Dichlorodiphenyltrichloroethane (DDT), first reported in 1991 in *An. gambiae s.s.* populations in Côte d’Ivoire [15], has spread within the species complex within SSA, including to Sierra Leone [4, 14]. Although some studies have indicated that ITNs remain effective for providing protection in areas with pyrethroid resistant vectors [16–19], other studies suggest that in areas where there is high levels of *kdr* in local vector populations, pyrethroid treated ITNs or pyrethroid-based IRS products failed to control the *An. gambiae s.l.* population [20, 21]. The new generation ITNs incorporating PBO or dual active ingredients have been shown to be more effective against pyrethroid resistant mosquitoes [22].

To better guide deployment of vector control interventions in Sierra Leone, the NMCP in collaboration with PMI VectorLink project assessed the susceptibility of malaria vectors to common insecticides used in malaria control as well as the distribution of molecular markers of insecticide resistance between 2018 and 19. This study investigated the status of phenotypic resistance to insecticides in the pyrethroid, organophosphate, carbamate, neonicotinoid, and pyrrole classes of insecticides and determined the occurrence and frequency of molecular mechanisms for pyrethroid, organophosphate and carbamate resistance. The aims were to characterize resistance of the *An. gambiae* complex mosquitoes to insecticides currently in use and under consideration for use in IRS and ITNs, to inform decision making for optimizing vector control.

## Methods

### Sampling area

**Fig. 1:**
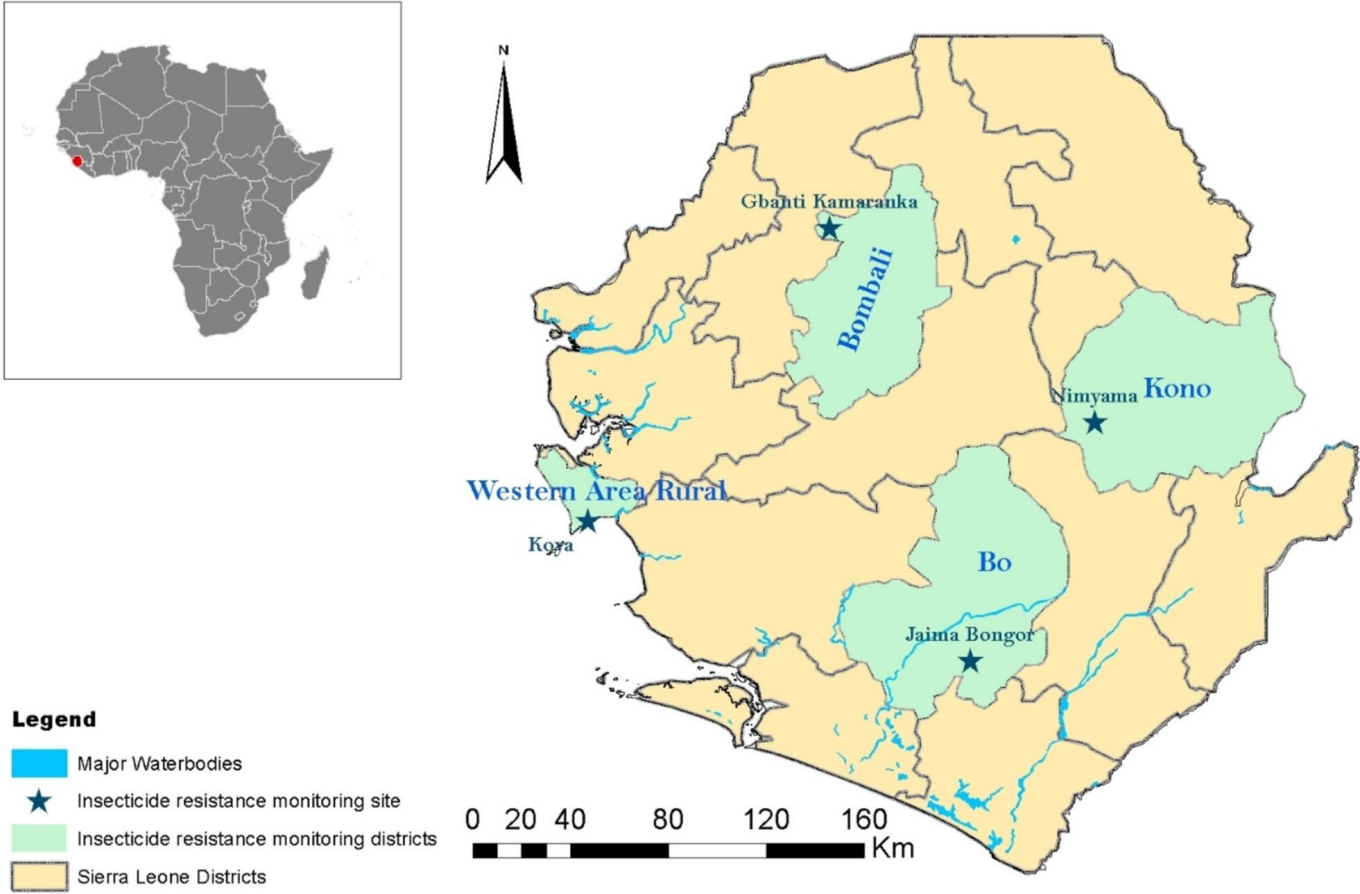
Insecticide and entomological monitoring sites in Sierra Leone

Sierra Leone is located on the west coast of Africa, bordered on the north and east by Guinea, on the south by Liberia, and opens into the Atlantic Ocean to the West. The study was conducted in four sites in four districts (Bo, Bombali, Kono and Western Rural Area) representing the different geographical regions of Sierra Leone. Bo represents the southern region; Bombali the northern; Kono the eastern; and Western Area Rural the west. All the districts lie within the rainforest belt vegetation with Western Area Rural having a coastal ecosystem with brackish water due to the presence of Atlantic Ocean. The rainfall pattern is similar in all sites, beginning in May and ending in October. The dry season begins in November through to April. In each district, mosquitoes were collected from one rural chiefdom: Jaima Bongor in Bo, Gbanti Kamaranka in Bombali, Nimyama in Kono and Koya in Western Area Rural (Fig. 1). The four sites are also where the PMI VectorLink project in Sierra Leone has been doing comprehensive entomological monitoring activities since 2018.

### Insecticide susceptibility tests

World Health Organization (WHO) tube tests [23] were used, and Centers for Disease Control and Prevention (CDC) bottle bioassays [24] were performed to assess the susceptibility of *An. gambiae* to the most common insecticides used in ITNs and IRS in Sierra Leone. The WHO impregnated test papers at diagnostic doses (1x) of pyrethroids (alpha-cypermethrin (0.05%), deltamethrin (0.05%) and permethrin (0.75%), pirimiphos methyl (0.25%) and bendiocarb (0.1%) were used to assess mosquito susceptibility. The WHO tube method was used to test *An. gambiae* susceptibility to pirimiphos methyl (0.25%) in all four sites and to bendiocarb (0.1%) in all sites except in Bombali, where insufficient mosquitoes were collected. The CDC bottle assay was used to assess the intensity of resistance to pyrethroids.

Larvae and pupae of *Anopheles* mosquitoes were collected from different breeding sites, mainly from temporary rain pools, in and around the sentinel sites up to a radius of 2 kms and reared to adults at the Vector-Borne Disease Insectary and Laboratory (VBDIL) in Makeni, Bombali District. The larvae collection period covered the rainy season (August to October) and dry season (November to February). Mosquitoes were morphologically identified at the adult stage using Gillies key [25] and only *An. gambiae* aged between 2 and 5 days old were used for the susceptibility tests. Tests were conducted at the VBDIL in Makeni at standard temperature of 26 ± 2 and humidity 80% ± 10 for all mosquitoes from the four districts: Bo, Bombali, Kono and Western Rural Area. The number of dead and alive mosquitoes in both the exposure and the control tubes were recorded after a 24 h post-exposure holding period. Given the slow-acting nature of clothianidin, post-exposure mortalities were scored every 24 h for up to five days or until 100% mortality was recorded, whichever came first.

The CDC bottle bioassay was also used to assess susceptibility of *An. gambiae* to chlorfenapyr. Two-to-five-day old *An. gambiae* reared from larvae were exposed to 250 ml Wheaton bottles treated with a diagnostic concentration of 100 µg/bottle chlorfenapyr. For clothianidin, the bottles were coated with 2% clothianidin. Tests with *An. gambiae* Kisumu strain as positive controls and negative controls without insecticide were run in parallel. Female *Anophele*s mosquitoes were introduced in batches of 20–25 in each replicate. After a 60 min exposure period, mosquitoes were released into clean cages and then gently aspirated into labelled paper cups covered with untreated netting and provided with 10% sugar solution. Knock-down was recorded 60 min after the start of the test, while mosquitoes were still in the bottle. Mortality was recorded every 24 h for up to five days or until 100% mortality was recorded, whichever came first.

### Synergist, and intensity assays of pyrethroids

First, mosquitoes were exposed to treated WHO papers at diagnostic doses (1x) of pyrethroids (alpha-cypermethrin (0.05%), deltamethrin (0.05%) and permethrin (0.75%)) with and without piperonyl butoxide 4% (PBO) synergist using the WHO procedure [23]. Adult *An. gambiae* mosquitoes aged 2–5 days old were pre-exposed to PBO for an hour followed by exposure to either deltamethrin, permethrin and alpha-cypermethrin impregnated papers (1x) in WHO tube tests. The number of dead and alive mosquitoes in both the exposure and the control tubes were recorded after a 24 h post-exposure holding period. The mortality estimates in the insecticide-only group, without pre-exposure to PBO, at diagnostic doses were also taken as 1 × in the intensity assays.

The CDC bottle bioassay [24] was used to assess the intensity of resistance to pyrethroids above the diagnostic dose. *Anopheles gambiae* adult mosquitoes were exposed to alpha-cypermethrin and deltamethrin at 62.5 (5x) and 125 (10x) µg/bottle. Permethrin was tested at a dose of 107.5 (5x) and 215 (10x) µg/bottle. These intensities were estimated in all sites except Kono, where the number of larvae collected was only enough to measure the intensity of alpha-cypermethrin resistance.

### Molecular species identification

A subset of 200 mosquitoes out of 5326, composed of those that survived or died following insecticide exposures, and representing all the districts that were tested were randomly sampled for molecular analysis. Genomic DNA was extracted from each mosquito sample via the Livak method [26] and stored at -20ºC following extraction. Identification of members of the *An. gambiae* complex was done using SINE polymerase chain reaction (PCR) that allows identification of *An. gambiae s.s., An. coluzzii* and *An. arabiensis* [27]. In coastal sites where other species such as *Anopheles melas* were suspected, the PCR-restriction fragment length polymorphism (PCR–RFLP) protocol described by Scott et al*.* [28] was used. PCR products were run via electrophoresis through a 1.5% agarose gel with Midori Green^®^ (Gene flow, UK) and visualized using ultraviolet light.

### Molecular characterization of resistance mechanisms

The subset samples above were also screened for the presence of the 1014F, 1014S, N1575Y, Ace-1G119S mutations using TaqMan assays [29–31]. Genotypes were scored from scatter plots of results produced by the Mx3005 v4.10 software. Three positive samples of known genotypes for each of the alleles were used as positive controls while distilled water was used as negative control for each of the experiments as described in the protocols ([29–32].

### Data analysis

Mortality was calculated by dividing the number of dead mosquitoes following exposure by total number exposed for each insecticide. Mortalities were corrected using Abbott’s formula if the mortality of control mosquitoes exposed to solvent only was ≥ 5% and < 20%. Tests were discarded and repeated if control mortalities were ≥ 20% [33]. Test results were also discarded and repeated if the mortality of mosquitoes exposed to PBO only was > 10%.

Susceptibility levels of *An. gambiae* were evaluated based on the WHO criteria of test mortality [23]: corrected mortality of 98–100% after 24–120 h post exposure indicated susceptibility; corrected mortality, > 90% but < 98%, indicated the existence of possible resistance; and mortality of < 90% indicated the presence of resistant individuals in the vector population.

Genotype frequencies per species per site was calculated as the relative frequency of the homozygote resistant and heterozygote resistant individuals. The allelic frequencies of L1014F, L1014S, N1575Y and *ace-1* were calculated as follows: F(R) = [2RR + RS]/[2(RR + RS + SS)]. The Hardy–Weinberg equation was used to calculate the expected genotype frequency of L1014F, L1014S, N1575Y and *ace-1* in *An. gambiae s.s.* and *An. coluzzii.* The expected and observed genotype frequencies were compared using Pearson’s Chi-squared tests in Microsoft Excel 2016 to determine statistical significance of differences and estimate inbreeding co-efficient (F_IS_) and STATA-SE12 was used to generate confidence intervals for the resistance allelic frequency distributions.

## Results

### Insecticide susceptibility and intensity assays

**Fig. 2:**
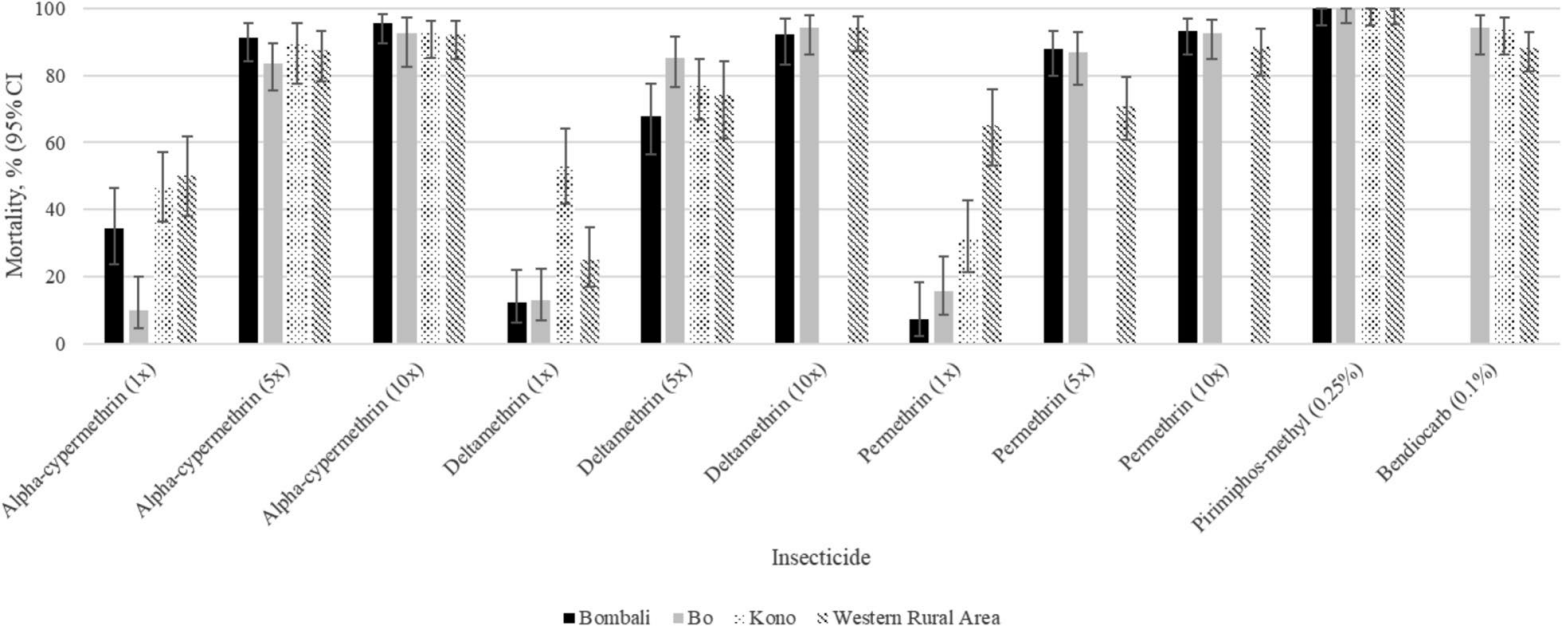
Susceptibility status of *An. gambiae* to different concentrations (1x, 5x, and 10x) of pyrethroids and diagnostic doses of pirimiphos-methyl (0.25%) and bendiocarb (0.1%) *Missing bars represent tests not done due to the insufficient numbers of mosquitoes collected*.*

A total of 5,326 *An. gambiae* were successfully reared to adults and exposed to different insecticides (supplementary material Table 1). *Anopheles gambiae* from all sampling sites were resistant to the diagnostic dose of deltamethrin, permethrin and alpha-cypermethrin (Fig. 2, supplementary material Table 1). The 24 h post-exposure mortality ranged from 7% in Bombali to 65% in Western Area Rural for permethrin; from 12% in Bombali to 53% in Kono for deltamethrin; and from 10% in Bo to 50% in Western Area Rural for alpha-cypermethrin (Fig. 2). *Anopheles gambiae* s.l. was fully susceptible to pirimiphos-methyl in all sites. For bendiocarb, there was resistance in Western Area Rural with 88% mortality and possible resistance in Bo and Kono with 94% mortality. Resistance intensity to alpha-cypermethrin, deltamethrin and permethrin was high at all sites, with mortality rates of *An. gambiae* below 98% at 10 times (10x) the diagnostic dose (Fig. 2). Mortality was below 90% at five times (5x) the diagnostic dose for all the three pyrethroids except for alpha-cypermethrin in Bombali (Fig. 2).

**Table 1:** Mean percent mortality of *An. gambiae* by holding time post exposure to chlorfenapyr and clothianidin

	Holding time				
Mean mortality	24 h	48 h	72 h	96 h	120 h
Chlorfenapyr	35.9	95.8	99.2	99.5	100
Clothianidin	94.1	98.5	99.7	99.7	100
Test	χ^2^ = 272.5	χ^2^ = 4.8	χ^2^ = 0.8	χ^2^ = 0.2	NA
P value	p = < 0.0001	p = 0.028	p = 0.273	p = 0.644	NA

**Fig. 3:**
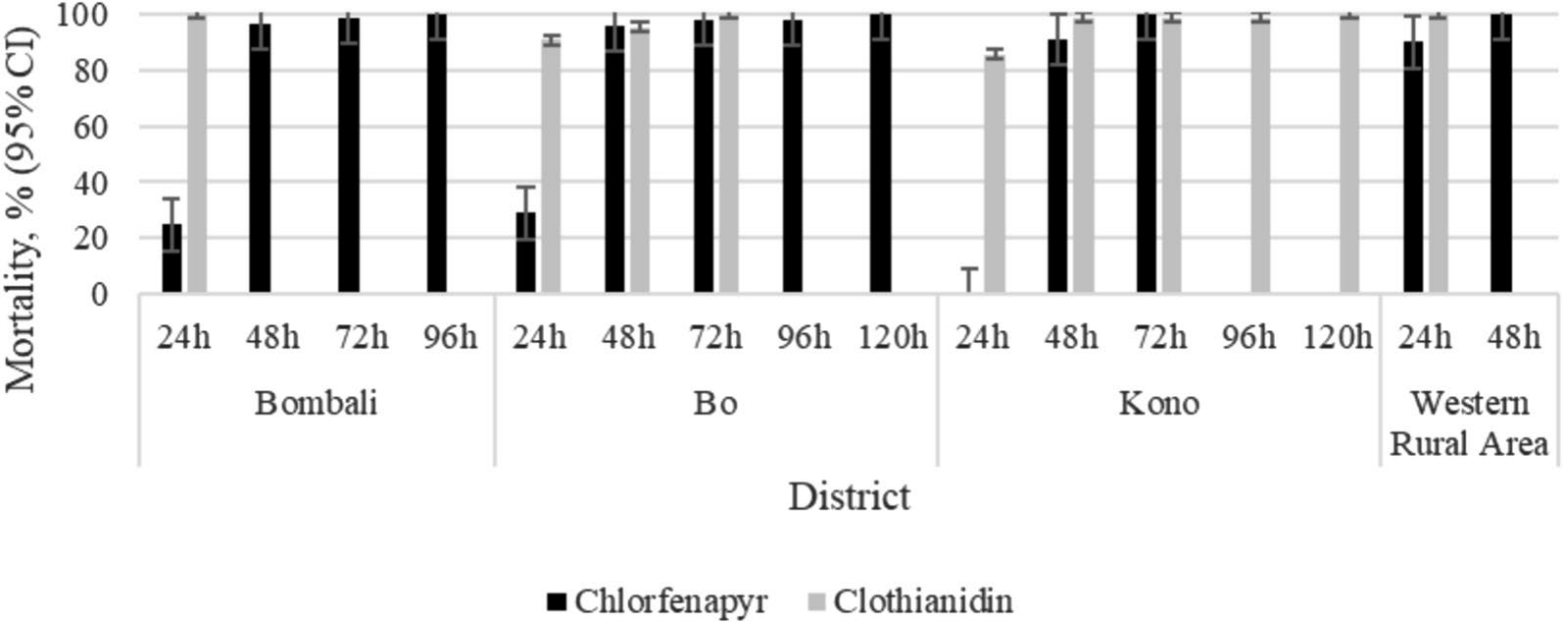
Mortality of *An. gambiae* to chlorfenapyr (100 µg/bottle) and clothianidin (2%)

*Anopheles gambiae* was susceptible to chlorfenapyr in all four sampling sites (Fig. 3). In all sites, over 98% of mosquitoes exposed to chlorfenapyr died after 72 h post-exposure with mortality reaching 100% after 120 h (4 days post exposure) (Fig. 3). *Anopheles gambiae* was also fully susceptible to clothianidin at all sites (Fig. 3). In Western Rural Area and Bombali, 100% mortality to clothianidin was recorded within the 24 h holding period. However, it took 72 h to achieve 100% mortality in Bo and 120 h in Kono (Fig. 3).

Overall, mosquitoes died faster after exposure to clothianidin than to chlorfenapyr. At 24 h holding time, the mean percent mortality was significantly higher (χ^2^ = 272.5; p < 0.0001) for clothianidin (94.1%) than for chlorfenapyr (35.9%) (Table 1). The difference narrowed at 48 h holding time, but it was still significantly higher (χ^2^ = 4.8; p = 0.028) for clothianidin than for chlorfenapyr. There was no difference at 72 h holding time (χ^2^ = 1.2; p = 0.273) and beyond (Table 1).

### Synergist assays

**Fig. 4:**
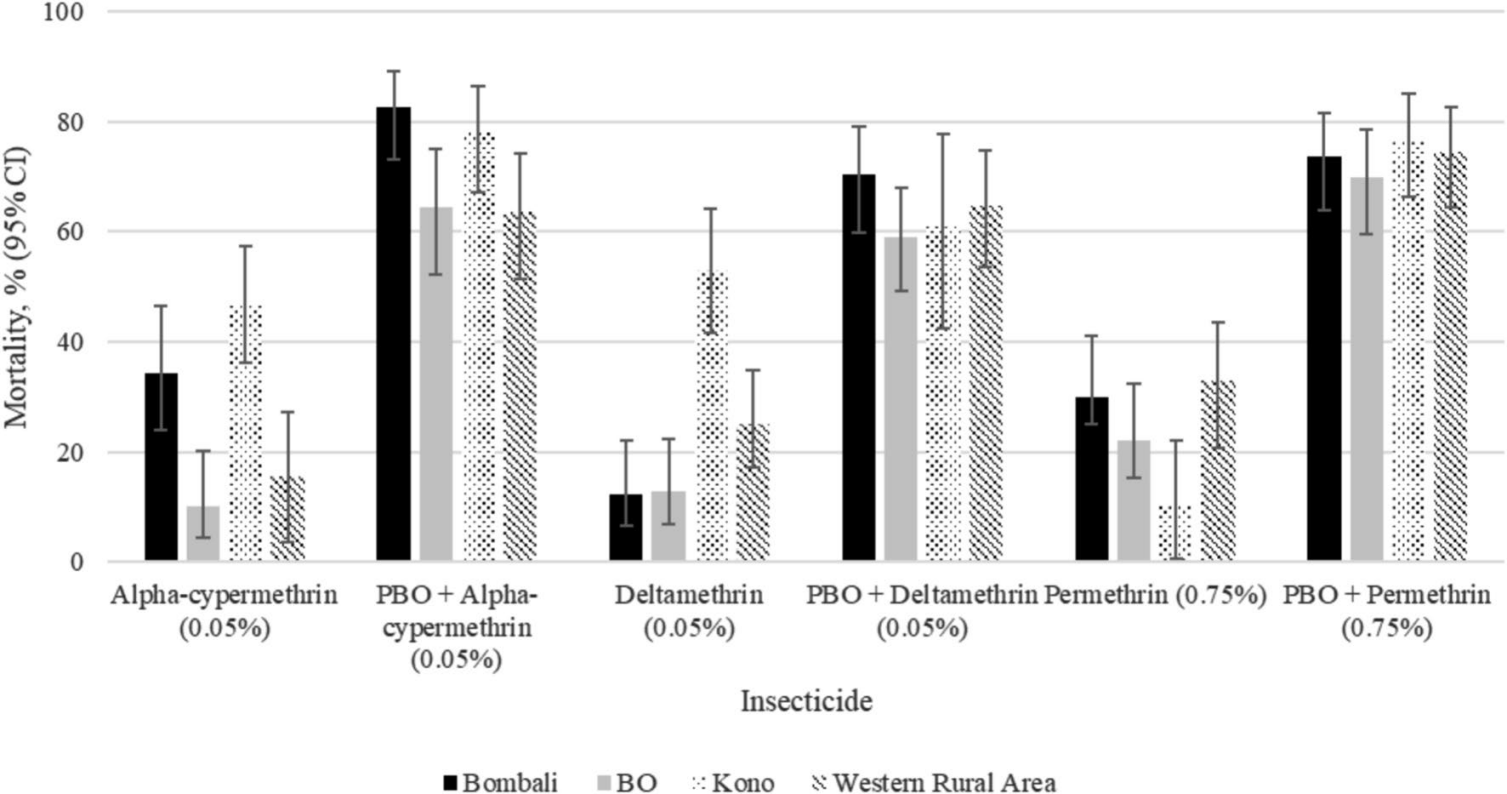
Mortality of *An. gambiae* to alpha-cypermethrin (0.05%), deltamethrin (0.05%) and permethrin (0.75%) without and post-PBO exposure

Exposure to 4% PBO increased mortality for each pyrethroid insecticide but full restoration of susceptibility above 98% was not achieved for any insecticides in any locations. The absolute increase after pre-exposure for deltamethrin ranged from 43.9% in Bombali to 52.9% in Bo. For alpha-cypermethrin, the absolute increase ranged between 41.7% in Bo and 56.0% in Kono. The absolute increase for permethrin was highest in Kono (66.3%) and lowest in Western Rural Area (41.5%) (Fig. 4).

### Molecular species identification

A total of 191 out of 200 (95.5%) mosquitoes were successfully identified to sibling species. *Anopheles gambiae s.s.* was dominant (81.7% 95% CI (75.3–86.7), followed by *An. coluzzii* (17.8%, 95% CI (12.8–24.1). One specimen was identified as a hybrid of *An. gambiae* and *An. coluzzii* 0.5%, 95% CI (0.03–3.3).

### Molecular characterization of resistance mechanisms

The frequency of the *kdr* L1014F resistance (r) allele was high, 0.98 (373/380); 95% CI: 0.94–0.99) whereas the *kdr* L1014S mutation frequency was low at 0.02 (8/382); 95% CI: 0.01–0.04) across all four assessment sites (Table 3). The wild-type allele for *kdr* L1014F was not detected in any of the *An. gambiae* or *An. coluzzii* specimens screened (Table 2, 3). The N1575Y mutation, which amplifies resistance conferred by L1014F and L1014S, was detected at a frequency of 0.089 (34/384) and were all in a heterozygous form (Table 2, 3). The difference in the frequency of the N1575Y mutation between *An. gambiae* (0.095) and *An. coluzzii* (0.059) was not statistically significant (p = 0.928). All 34 samples with the N1575Y mutation also carried the homozygous mutant allele L1014F. No deviation from the Hardy–Weinberg equilibrium and no deficiency of heterozygous individuals was detected at the L1014F, L1014S and N1575Y loci (Table 3. 4).

**Table 2:** Distribution of molecular markers of insecticide resistance by phenotype in Sierra Leone

		*Kdr-w*				*Kdr-e*				*Ace-1*				N1575Y			
District	Phenotype	FF	LF	NA	Total	LS	LL	NA	Total	SS	GG	NA	Total	NY	NN	NA	Total
Bo	Resistant	31	2		33	4	29		33	4	29		33	3	30		33
	Susceptible	17			17	1	15	1	17	1	16		17	2	15		17
Bombali	Resistant	31			31	3	28		31		31		31	8	23		31
	Susceptible	19		1	20		19	1	20	3	16	1	20	5	14		19
Kono	Resistant	27	2	2	31		30	1	31	3	27	1	31	4	27		31
	Susceptible	18	1		19	1	18		19	2	17		19	1	18		19
Western Rural Area	Resistant	31			31		31		31	12	19		31	8	23		31
	Susceptible	17	2		19		18	1	19	1	18		19	4	14	2	20
Total		**191**	**7**	**3**	**201**	**9**	**188**	**4**	**201**	**26**	**173**	**2**	**201**	**35**	**164**		**201**

Bold values are column totals

*NA* Not amplified, *LL* homozygous wild type, *LF* heterozygous resistant for *kdr-w*, *FF* homozygous resistant for *kdr-w*, *LS* heterozygous resistant for *kdr-e*, *GG* homozygous wild type, *SS* homozygous resistant for *Ace*-1, *NN* homozygous wild type for N1575Y, *NY* heterozygous resistant for N15756Y

**Table 3:** Allelic frequencies of L1014F and L1014S

Site	Species	L1014													
		N	LL	LF	FF	f (L1014F)	95% CI	F_IS_	N	LL	LS	SS	f(L1014S)	95% CI	F_IS_
Bo	*An. coluzzii*	17	0	1	16	0.97	(0.85–1.00)	− 0.03030	17	16	1	0	0.03	(0.00–0.15)	− 0.0303
	*An. gambiae*	30	0	1	29	0.98	(0.91–1.00)	− 0.01695	30	27	3	0	0.05	(0.01–0.14)	− 0.05263
Bombali	*An. coluzzii*	6	0	0	6	1.00	(0.74–1.00)	−	6	6	0	0	0.00	(0.00–0.27)	–
	*An. gambiae*	44	0	0	44	1.00	(96.0–1.00)	−	44	41	3	0	0.03	(0.01–0.10)	− 0.03529
Kono (Eastern)	*An. coluzzii*	4	0	1	3	0.88	(0.47–1.00)	− 0.14286	4	3	1	0	0.01	(0.00–0.53)	− 0.14286
	*An. gambiae*	42	0	2	40	0.98	(0.92–1.00)	− 0.02439	43	43	0	0	0.00	(0.00–0.04)	–
Western Rural Area	*An. coluzzii*	7	0	2	5	0.86	(0.57–0.98)	− 0.16667	7	7	0	0	0.00	(0.00–0.23)	–
	*An. gambiae*	40	0	0	40	1.00	(95.0–1.00)	–	40	40	0	0	0.00	(0.00–0.05)	–
Total		**190**	**0**	**7**	**183**	**0.98**	**(0.94–0.99)**	**− 0.01799**	**191**	**183**	**8**	**0**	**0.02**	**(0.01–0.04)**	**− 0.02139**

Bold values are column totals

*N* Number of mosquitoes tested, *LL* homozygous wild type, *LF* heterozygous resistant for *kdr-w*, *FF*  homozygous resistant for *kdr-w*, f (L1014F) = frequency of the L1014F resistant allele, *LS* heterozygous resistant for *kdr-e*, *SS* homozygous resistant for *kdr-e*; f (L1014S) = frequency of the L1014S resistant allele

F_IS_ Values > 0 indicate heterozygote deficiency, while values < 0 indicate heterozygote excess

**Table 4:** Allelic frequencies of N1575Y and G119S

Site	N1575Y								G119S						
	Species	N	NN	NY	YY	f(N1575Y)	95% CI	F_IS_	N	GG	GS	SS	f(G119S)	95% CI	F_IS_
Bo	*An. coluzzii*	17	16	1	0	0.03	(0.00–0.15)	− 0.03030	17	17	0	0	0.00	(0.00–0.10)	–
	*An. gambiae*	30	27	3	0	0.05	(0.01–0.14)	− 0.05263	30	25	0	5	0.17	(0.08–0.29)	1.000*
Bombali	*An. coluzzii*	6	4	2	0	0.17	(0.02–0.48)	− 0.20000	6	6	0	0	0.00	(0.00–0.26)	–
	*An. gambiae*	44	33	11	0	0.13	(0.06–0.21)	− 0.14286	44	41	0	3	0.07	(0.03–0.14)	1.000*
Kono	*An. coluzzii*	4	4	0	0	0.00	(0.00–0.37)	–	4	4	0	0	0.00	(0.00–0.37)	–
	*An. gambiae*	44	39	5	0	0.06	(0.02–0.13)	− 0.06024	43	38	0	5	0.12	(0.06–0.20)	1.000*
Western Rural Area	*An. coluzzii*	7	6	1	0	0.07	(0.00–0.34)	− 0.07692	7	7	0	0	0.00	(0.00–0.23)	–
	*An. gambiae*	40	29	11	0	0.14	(0.07–0.23)	− 0.15942	40	27	0	13	0.33	(0.22–0.44)	1.000*
Total		**192**	**158**	**34**	**0**	**0.09**	**(0.06–0.12)**	**− 0.09714**	**191**	**165**	**0**	**26**	**0.14**	**(0.01–0.17)**	**1.000**

Bold values are column totals

N = Numbe of mosquitoes tested; GG = homozygous wild type; GS = heterozygous resistant for *Ace*-1; SS = homozygous resistant for *Ace*-1; f (G119S) = frequency of the G119S resistant allele; NN = homozypous wild type for N1575Y; NY = heterozygous resistant for N15756Y; YY = homozygous resistant for N1575Y; f (N1575Y) = frequency of the N1575Y resistant allele

F_IS_ values > 0 indicate heterozygote deficiency, while values < 0 indicate heterozygote excess

The overall frequency of the G119S mutation was 0.14 (N = 52). No G119S mutation was found in *An. coluzzii* (Table 4). However, both deviation from the Hardy–Weinberg equilibrium and deficiency of heterozygotes was detected at the G119S locus of *An. gambiae s.s.* and was statistically significant (p < 0.0001) (Table 5). As all the samples were either homozygous or heterozygous to L1014F mutation, all the specimens with L1014S, G119S and N1575Y also had the L1014F resistance allele. All mosquitoes with L1014S did not have the G119S mutation and only one was carrying the N1575Y mutation. Seven *An. gambiae* s.l. specimens were found to be carrying three mutations: L1014F plus either L1014S or G119S, or L1014F plus G119S and N1575Y (Table 6).

**Table 5:** Genotype frequencies for the L1014F, L1014S, G119S, and N1575Y mutation in *An. gambiae* from Sierra Leone. Tests of Hardy–Weinberg Equilibrium (χ2) with corresponding P-values

Mutation	Site	N	Observed			χ2	P-Value
			RR	RS	SS		
L1014F	Bo (Southern)	50	0.960	0.040	0.000	0.0208	0.8853
	Bombali (Northern)	50	1.000	0.000	0.000	0.0000	1.0000
	Western Rural Area (Western)	50	0.960	0.040	0.000	0.0208	0.8853
	Kono (Eastern)	48	0.938	0.063	0.000	0.0499	0.8232
L1014S	Bo (Southern)	49	0.000	0.102	0.898	0.1416	0.7067
	Bombali (Northern)	50	0.000	0.060	0.940	0.0478	0.8269
	Western Rural Area (Western)	49	0.000	0.000	1.000	0.0000	1.0000
	Kono (Eastern)	49	0.000	0.020	0.980	0.0052	0.9425
G119S	Bo (Southern)	50	0.100	0.000	0.900	50.0000	< .00001
	Bombali (Northern)	50	0.060	0.000	0.940	50.0000	< .00001
	Western Rural Area (Western)	50	0.260	0.000	0.740	50.0000	< .00001
	Kono (Eastern)	49	0.102	0.000	0.898	49.0000	< .00001
N1575Y	Bo (Southern)	50	0.000	0.100	0.900	0.1385	0.7098
	Bombali (Northern)	50	0.000	0.260	0.740	1.1164	0.2907
	Western Rural Area (Western)	49	0.000	0.245	0.755	0.9540	0.3287
	Kono (Eastern)	50	0.000	0.100	0.900	0.1385	0.7098

**Table 6:** Multiple mutations in *An. gambiae*

Mutations	L1014F		L1014S		G119S		N1575Y	
Phenotype	Resistant	Susceptible	Resistant	Susceptible	Resistant	Susceptible	Resistant	Susceptible
L1014F	–		–	–	–	–	23	11
L1014S	6	2	–	–	–	–	1	0
G119S	19	7	0	–	–	–	6	0
N1575Y	23	11	1*	–	6*	–	–	–

^*^Also carry the L1014F mutation; 7 samples were with 3 types of mutations

Seven of the eight samples with the G119S mutation were also survivors of the exposure to bendiocarb. However, 68.2% (15/22) of the samples that were phenotypically resistant to bendiocarb were not carrying the G119S mutation. Ninety-five percent (19/20) of the samples susceptible to bendiocarb and pirimiphos-methyl were also not carrying the mutation (Table 7).

**Table 7:** Phenotypic resistance of *An. gambiae* to bendiocarb and allelic frequencies of G119S

Species	Phenotype	N	*G119S*			
			GG	GS	SS	*Odds ratio*
*An. gambiae* s.l	Resistant/Alive	22	15	0	7	7.7, p = 0.03
	Susceptible/Dead	20	19	0	1	Ref.
*Total*		42	34	0	8	

*GG* homozygous wild type, *GS* heterozygous resistant for *Ace*-1, *SS* homozygous resistant for *Ace*-1

## Discussion

*Anopheles gambiae s.s.* was the dominant malaria vector in Sierra Leone followed by *An. coluzzii*. Consistent with previous studies, they were the only species sampled in larval habitats [13]. In Sierra Leone, *An. gambiae* was highly resistant to the three pyrethroid insecticides tested with partial restoration of susceptibility following pre-exposure to PBO suggesting involvement of metabolic resistance mediated by P450 enzymes. The presence of survivors following exposure to 10 × the discriminating doses is indicative of the high intensity of resistance to pyrethroids. The observed range of enhancement of mortality due to pre-exposure to PBO was similar across the three pyrethroids tested.

This assessment also demonstrated the *kdr* L1014F resistance mutation to be present at high frequency, which is not surprising, considering the high level of phenotypic resistance to pyrethroids. Though not frequently identified in previous samples, this report is a first of *kdr* L1014S and N1575Y mutations in Sierra Leone. The N1575Y mutation, located within the linker between domains III-IV in the voltage-gated sodium channel (*Vgsc)* and believed to have a synergistic effect on pyrethroid and DDT resistance when combined with the L1014F mutation was previously reported from Burkina Faso, Benin, Cameroon and Côte d’Ivoire [32, 34–36].

The detection of the N1575Y mutation in Sierra Leone requires more investigation to better characterize its expected synergistic relationship with 1014F *kdr*. The presence of this additional mechanism that could further reduce insecticide efficacy in the already pyrethroid-resistant mosquitoes in Sierra Leone is concerning. Thus, monitoring of N1575Y should continue in order to understand its contribution to insecticide resistance among local vectors. This resistance mechanism could spread very rapidly [32] and threaten the malaria vector control efforts in Sierra Leone which had relied primarily on the distribution of pyrethroid-treated ITNs. This finding also provides evidence for justifying the need to move to the distribution of non-pyrethroid nets.

As the effect of PBO exposure was similar across the three pyrethroids tested, nets treated with any of the pyrethroids plus PBO might have similar effects and can be used as alternative tools in the national Sierra Leone Insecticide Resistance Management Plan. Indeed, NMCP distributed PermaNet 3.0 (deltamethrin + PBO) and Olyset Plus (permethrin + PBO) in the 2020 mass net campaign and these contributed to malaria reduction in Sierra Leone [37]. These data not only yielded the evidence that PBO nets could provide better protection than pyrethroid nets without PBO in Sierra Leone, but also demonstrated that in the absence of non-pyrethroid ITNs available at the time of this assessment, Sierra Leone had the flexibility to choose PBO ITNs treated with any of the three pyrethroids. Sierra Leone was the world’s first country to provide population-level coverage nationally with next-generation PBO ITNs during a mass distribution campaign and evaluations to measure their durability and performance are underway. The NMCP has now incorporated these data and subsequent data on insecticide resistance to procure IG2 nets for 2023 mass ITN campaign.

*Anopheles gambiae* was susceptible to pirimiphos-methyl (organophosphate), chlorfenapyr (pyrrole) and clothianidin (neonicotinoid). However, there was an indication of possible resistance to bendiocarb (carbamate). As the NMCP prepared to implement IRS in Bo and Bombali districts in May/June 2021 at the start of the rainy season, the findings from this assessment provided critical data that guided the selection of an appropriate insecticide for the IRS program. Thus, clothianidin was selected for IRS because vectors were fully susceptible to it. There was no phenotypic resistance to pirimiphos-methyl and low-level resistance to bendiocarb was reported. However, carbamates and organophosphates are known to share the *ace*-*1* pathway as a resistance mechanism [38]. With the reported phenotypic resistance to bendiocarb and the reported prevalence of the G119S mutation at 13.6% in the vector population, this mutation needs close monitoring to guide future IRS in Sierra Leone. Moreover, the combination of IRS using pirimiphos-methyl and PBO nets have been suggested to be antagonistic [39]. Thus, with the mass distribution of PBO nets that occurred in Sierra Leone in 2020 and continued PBO ITN distribution through some routine channels, and considering this potential antagonism between vector control interventions, caution should be used in the selection of pirimiphos-methyl for IRS. The susceptibility of the vector to chlorfenapyr also suggests that its use for IRS or as part of ITN treatment is a possibility when it becomes available.

The presence of three or more *ace-1* alleles in *An. gambiae s.s.* mosquitoes was previously documented in several countries in West Africa [40–42]. However, prior to the present assessment, little information was available for Sierra Leone. Although the G119S mutation is indicative of the phenotypic resistance to bendiocarb in this study, the high number of wild types that survived exposure to bendiocarb indicates involvement of other resistance mechanisms in Sierra Leone. The relatively low frequency of the G119S mutation (0.14; N = 26) was consistent with the low phenotypic resistance to bendiocarb and full susceptibility to pirimiphos-methyl observed in this assessment.

The absence of the G119S mutation in *An. coluzzii* cannot be explained by geographic variation, as both species were sympatric and collected from the same breeding sites. This finding is different from previous reports from West Africa, which observed this mutation in higher frequencies in both species: 0.32 in *An. gambiae s.s.* and 0.04 in *An. coluzzii* from Burkina Faso [41], 0.31 in *An. gambiae s.s*. and 0.35 in *An. coluzzii* from Côte d’Ivoire [43, 44], and 0.24 in *An. gambiae s.s.* and 0.04 in *An. coluzzii* from Ghana [40]. The G119S mutation has been associated with a high fitness cost in *Culex pipiens* populations [38, 45]. Indications of a high fitness cost were also similar in *An. gambiae s.s.* as the frequency of the *ace‐1* mutation in mosquito populations was observed to decline rapidly after a few generations without selection pressure from organophosphates or carbamates [41, 46]. In Burkina Faso, an excess of heterozygous genotypes in S form populations likely indicated that a fitness cost is associated with the mutation when present in a homozygous form. However, the findings from this assessment were different, such that all the *ace-1* G119S mutations were detected in the homozygous form with complete deficiency of heterozygotes and in significant deviation from the Hardy–Weinberg equilibrium. Nevertheless, the phenomenon of heterozygote deficiency in the G119S mutation is not new, as other studies have reported such a deficit in some sites in Cameroon [42] but does suggest a need for further exploration.

In the study by Elanga-Ndille et al. [42] in Cameroon, each sequenced individual specimen possessed at least two distinct *ace-1* resistant alleles and one susceptible allele. This was a possible explanation for why most mosquitoes that remained alive after carbamate exposure were genotyped as homozygote resistant with a lack of heterozygotes: mosquitoes with two copies of the gene seemed to have three resistant alleles vs. only one susceptible allele. It is hypothesized that this duplication decreases the fitness cost associated with the resistant genotype [46, 47] thereby hindering carbamate-based vector control strategies [36]. Since no mosquitoes survived the pirimiphos-methyl bioassays and the few that survived the bendiocarb exposure were not tested separately, the link between the G119S mutation and phenotypic resistance to carbamates and organophosphates was not investigated. However, six of the 13 mosquitoes from the bioassay with bendiocarb were carrying the *Ace*-1 G119S mutation homozygous allele.

The *ace-1* G119S mutation (associated with a low level of resistance to carbamate insecticides) was also present in *An. gambiae s.s*. but not in *An. coluzzii*. The G119S *ace-1* mutation was not associated with pirimiphos-methyl resistance. However, further bioassays and screening for the *ace-1* resistance allele on a wider scale would be required to understand the implications of the current status of the *ace-1* mutation for the efficacy of organophosphate insecticide use in vector control in Sierra Leone.

## Conclusion

The resistance profile of *An. gambiae* in Sierra Leone is relatively similar to that of other West African countries. It is characterized by a high level of pyrethroid resistance and an almost fixed L1014F mutation coupled with evolving L1014S, G119S and N1575Y mutations. Sierra Leone should continue to monitor the effects of the 2020 distribution of PBO nets on insecticide resistance evolution in mosquito vectors and the resulting epidemiological impacts associated with malaria burden reduction. Additionally, given the high frequency of the L1014F mutation, the emergence of the N1575 mutation and the modest increase in the susceptibility of the vector after pre-exposure to PBO shown in this assessment, it would be prudent for the NMCP to maximize the use of nets treated with non-pyrethroids for future mass distribution cycles and routine distribution channels. This assessment has demonstrated that neonicotinoid- and pyrrole-based insecticides show promising results in terms of vector susceptibility, but further assessment of their residual bio-efficacy and other implementation-related factors such as cost, and community acceptance is required to select insecticides for evidence-based vector control. Taken as a whole, this assessment has generated important information on the resistance profile of the main malaria vectors against the most common insecticides used in Sierra Leone. These results have formed the basis for further entomological studies and supported the NMCP of Sierra Leone in its development of insecticide resistance monitoring and management strategies.

## Supplementary Information

Supplementary material 1. Table 8: Number of mosquitoes tested for susceptibility against pyrethroids with and without PBO, carbamates, organophosphates and neonicotinoids insecticides and percent mortality with 95% confidence intervals, between 2018 and 2019.

## Data Availability

The datasets used and/or analyzed during the current study are available from the corresponding author on reasonable request.

## Abbreviations

ANC: Antenatal care
*ace-1*: Acetylcholinesterase 1
CDC: Centers for Disease Control and Prevention
DDT: Dichlorodiphenyltrichloroethane
DNA: Deoxyribonucleic acid
EPI: Expanded programme on immunization
IRS: Indoor residual spraying
ITN: Insecticide-treated nets
*kdr*: Knock-down resistance
NMCP: National Malaria Control Programme
PBO: Piperonyl butoxide
PCR: Polymerase chain reaction
PMI: US President’s Malaria Initiative
RFLP: Restriction fragment length polymorphism
*Vgsc*: Voltage-gated sodium channel
WHO: World Health Organization
