# Supplementary material for: Characterization of insecticide resistance mechanisms in the Anopheles gambiae population of Sierra Leone
Source: Malar J. 2025 Mar 13;24:80. doi: 10.1186/s12936-025-05267-z (PMC11907884; doi:10.1186/s12936-025-05267-z)
Supplement: Supplementary file 1 — Supplementary material 1. Table 8: Number of mosquitoes tested for susceptibility against pyrethroids with and without PBO, carbamates, organophosphates and neonicotinoids insecticides and percent mortality with 95% confidence intervals, between 2018 and 2019. [file 12936_2025_5267_MOESM1_ESM.docx]

**Supplementary Material**

Table 1: Number of mosquitoes tested for susceptibility against pyrethroids with and without PBO, carbamates, organophosphates and neonicotinoids insecticides and percent mortality with 95% confidence intervals, between 2018 and 2019.

|  |  |  |  | 95% CI | |
| --- | --- | --- | --- | --- | --- |
| **Bombali** | Total exposed | Dead | Mortality | lower | upper |
| Alpha-cypermethrin (1x) | 73 | 25 | 34.2 | 23.8 | 46.4 |
| Alpha-cypermethrin (5x) | 115 | 105 | 91.3 | 84.2 | 95.5 |
| Alpha-cypermethrin (10x) | 114 | 109 | 95.6 | 89.6 | 98.4 |
| Alpha-cypermethrin + PBO | 97 | 80 | 82.5 | 73.1 | 89.2 |
| Deltamethrin (1x) | 81 | 10 | 12.3 | 6.4 | 22 |
| Deltamethrin (5x) | 81 | 55 | 67.9 | 56.5 | 77.6 |
| Deltamethrin (10x) | 77 | 71 | 92.2 | 83.2 | 96.8 |
| Deltamethrin + PBO | 91 | 64 | 70.3 | 59.7 | 79.2 |
| Permethrin (1x) | 55 | 4 | 7.3 | 2.4 | 18.4 |
| Permethrin (5x) | 108 | 95 | 88 | 79.9 | 93.2 |
| Permethrin (10x) | 104 | 97 | 93.3 | 86.1 | 97 |
| Permethrin + PBO | 102 | 75 | 73.5 | 63.7 | 81.5 |
| Pirimiphos-methyl (0.25%) | 93 | 93 | 100 | 95.1 | 100 |
| Chlorfenapyr | 84 | 84 | 100 | 94.6 | 100 |
| Clothianidin | 99 | 99 | 100 | 95.3 | 100 |
|  |  |  |  |  |  |
| **Bo** |  |  |  |  |  |
| Alpha-cypermethrin (1x) | 70 | 7 | 10 | 4.5 | 20.1 |
| Alpha-cypermethrin (5x) | 122 | 102 | 83.6 | 75.6 | 89.5 |
| Alpha-cypermethrin (10x) | 67 | 62 | 92.5 | 82.7 | 97.2 |
| Alpha-cypermethrin + PBO | 73 | 47 | 64.4 | 52.2 | 75 |
| Deltamethrin (1x) | 85 | 11 | 12.9 | 6.9 | 22.4 |
| Deltamethrin (5x) | 96 | 82 | 85.4 | 76.4 | 91.5 |
| Deltamethrin (10x) | 86 | 81 | 94.2 | 86.3 | 97.8 |
| Deltamethrin + PBO | 112 | 66 | 58.9 | 49.2 | 68 |
| Permethrin (1x) | 77 | 12 | 15.6 | 8.7 | 26 |
| Permethrin (5x) | 83 | 72 | 86.7 | 77.1 | 92.9 |
| Permethrin (10x) | 95 | 88 | 92.6 | 84.9 | 96.7 |
| Permethrin + PBO | 96 | 67 | 69.8 | 59.4 | 78.5 |
| Pirimiphos-methyl (0.25%) | 102 | 102 | 100 | 95.5 | 100 |
| Bendiocarb (0.1%) | 85 | 80 | 94.1 | 86.2 | 97.8 |
| Chlorfenapyr | 97 | 97 | 100 | 95.3 | 100 |
| Clothianidin | 86 | 86 | 100 | 94.7 | 100 |
|  |  |  |  |  |  |
| **Kono** |  |  |  |  |  |
| Alpha-cypermethrin (1x) | 92 | 43 | 46.7 | 36.4 | 57.4 |
| Alpha-cypermethrin (5x) | 56 | 50 | 89.3 | 77.4 | 95.6 |
| Alpha-cypermethrin (10x) | 106 | 98 | 92.5 | 85.2 | 96.4 |
| Alpha-cypermethrin + PBO | 78 | 61 | 78.2 | 67.1 | 86.4 |
| Deltamethrin (1x) | 81 | 43 | 53.1 | 41.7 | 64.1 |
| Deltamethrin (5x) | 91 | 70 | 76.9 | 66.7 | 84.8 |
| Deltamethrin (10x) | 0 | 0 | 0 | 0 | 0 |
| Deltamethrin + PBO | 31 | 19 | 61.3 | 42.3 | 77.6 |
| Permethrin (1x) | 77 | 24 | 31.2 | 21.4 | 42.9 |
| Permethrin (5x) | 0 | 0 | 0 | 0 | 0 |
| Permethrin (10x) | 0 | 0 | 0 | 0 | 0 |
| Permethrin + PBO | 86 | 66 | 76.7 | 66.2 | 84.9 |
| Pirimiphos-methyl (0.25%) | 88 | 88 | 100 | 94.8 | 100 |
| Bendiocarb (0.1%) | 94 | 88 | 93.6 | 86.1 | 97.4 |
| Chlorfenapyr | 100 | 100 | 100 | 95.4 | 100 |
| Clothianidin | 84 | 84 | 100 | 94.5 | 100 |
|  |  |  |  |  |  |
| **Western Area** |  |  |  |  |  |
| Alpha-cypermethrin (1x) | 64 | 32 | 50 | 38.1 | 61.9 |
| Alpha-cypermethrin (5x) | 88 | 77 | 87.5 | 78.3 | 93.3 |
| Alpha-cypermethrin (10x) | 105 | 97 | 92.4 | 85.1 | 96.4 |
| Alpha-cypermethrin + PBO | 74 | 47 | 63.5 | 51.5 | 74.2 |
| Deltamethrin (1x) | 100 | 25 | 25 | 17.1 | 34.8 |
| Deltamethrin (5x) | 62 | 46 | 74.2 | 61.3 | 84.1 |
| Deltamethrin (10x) | 102 | 96 | 94.1 | 87.1 | 97.6 |
| Deltamethrin + PBO | 85 | 55 | 64.7 | 53.5 | 74.6 |
| Permethrin (1x) | 72 | 47 | 65.3 | 53.1 | 75.9 |
| Permethrin (5x) | 100 | 71 | 71 | 60.9 | 79.4 |
| Permethrin (10x) | 96 | 85 | 88.5 | 80 | 93.9 |
| Permethrin + PBO | 98 | 73 | 74.5 | 64.5 | 82.5 |
| Pirimiphos-methyl (0.25%) | 96 | 96 | 100 | 95.2 | 100 |
| Bendiocarb (0.1%) | 129 | 114 | 88.4 | 81.2 | 93.1 |
| Chlorfenapyr | 89 | 89 | 100 | 94.8 | 100 |
| Clothianidin | 96 | 96 | 100 | 95.2 | 100 |
| **Total** | **5,326** |  |  |  |  |
